# The modified Glasgow prognostic score is a reliable predictor of oncological outcomes in patients with rectal cancer undergoing neoadjuvant chemoradiotherapy

**DOI:** 10.1038/s41598-023-44431-w

**Published:** 2023-10-10

**Authors:** Atsushi Shimada, Takeru Matsuda, Ryuichiro Sawada, Hiroshi Hasegawa, Kimihiro Yamashita, Hitoshi Harada, Naoki Urakawa, Hironobu Goto, Shingo Kanaji, Taro Oshikiri, Yoshihiro Kakeji

**Affiliations:** 1 Division of Gastrointestinal Surgery, Department of Surgery, Kobe University Graduate School of Medicine, Kobe, Japan; 2 Division of Minimally Invasive Surgery, Department of Surgery, Kobe University Graduate School of Medicine, 7-5-2 Kusunoki-chou, Chuo-ku, Kobe, 650-0017 Japan

**Keywords:** Gastroenterology, Oncology

## Abstract

There has been no reliable marker for predicting oncological outcomes in patients with locally advanced rectal cancer (LARC) undergoing neoadjuvant chemoradiotherapy (NACRT). We retrospectively analyzed 73 patients with LARC who underwent curative surgery after NACRT. The modified Glasgow prognostic score (mGPS) was assessed after NACRT, and clinical outcomes were compared between the high (mGPS = 1 or 2; n = 23) and low (mGPS = 0; n = 50) groups. Body mass index was significantly higher in the low mGPS group. The 5-year disease-free survival (DFS) rate was significantly worse in the high mGPS group than that in the low mGPS group (36.7% vs. 76.6%, p = 0.002). Univariate and multivariate analyses of DFS revealed that mGPS was the most significant predictor (p < 0.001). mGPS appears to be a reliable predictor of oncological outcomes in patients with LARC undergoing NACRT.

## Introduction

In Western countries, the standard treatment for stage II–III locally advanced rectal cancer (LARC) is neoadjuvant chemoradiotherapy (NACRT), followed by total mesorectal excision (TME)^1–4^. It offers improvements in disease-free survival (DFS) and overall survival (OS), and the “watch-and-wait” approach is an alternative treatment when a clinical complete response is achieved^5,6^. However, NACRT does not benefit all patients^7–9^. Although good responders to NACRT may benefit from improved local control and organ preservation, poor responders suffer considerable side-effects and might lose their best opportunity for surgery. Therefore, a predictor of response to NACRT is necessary. Although the most reliable marker is the pathological response to NACRT, it can only be assessed postoperatively.

A systemic inflammatory response is implicated in the prognosis of patients with various cancers^10,11^. The modified Glasgow prognostic score (mGPS), consisting of C-reactive protein (CRP) and albumin levels, reflects the patient’s systemic inflammation and nutritional levels^12^. Several investigators have reported a significant association between the mGPS and patient prognosis in several types of cancer, including colorectal cancer (CRC)^13,14^. However, whether the mGPS correlates with the prognosis of patients with LARC undergoing NACRT remains unclear. This study aimed to investigate the clinical significance of the mGPS in predicting the oncological outcomes of patients with LARC undergoing NACRT.

## Materials and methods

### Patients

We retrospectively reviewed the data of patients with LARC who underwent curative surgery after NACRT at the Kobe University Hospital between November, 2005 and August, 2020. A total of 77 patients met the following criteria: (1) histologically proven adenocarcinoma, (2) lower tumor margin below the peritoneal reflection, and (3) cT3/4 or cN+ disease without distant metastasis. Although tumors were classified according to the American Joint Committee on Cancer TNM Classification of Malignant Tumors^15^, lateral pelvic lymph nodes (LLNs) were regarded as regional lymph nodes. Therefore, only patients with clinically positive LLNs were included in the present study. Four patients lacking blood data were excluded. Therefore, 73 patients were included in the analysis.

This study was approved by the institutional review board (IRB) of Kobe University (approval no. B210279). Due to the retrospective nature of the study, the IRB of Kobe University waived the need of obtaining informed consent. All methods were performed in accordance with the relevant guidelines and regulations.

### Treatment strategy

NACRT consisted of a total radiation dose of 45 or 50.4 Gy and oral 5-fluorouracil (5-FU)-based chemotherapy, as previously described^16^. Radiotherapy was delivered in 25 or 28 fractions for 5 weeks, covering the lateral pelvic area in the radiation target volume. Concurrent chemotherapy was initiated on day 1 of radiotherapy. Imaging and blood examinations were performed 4–6 weeks after NACRT and before surgery. Surgery was performed 6–8 weeks after NACRT. TME was performed in all patients through open or laparoscopic surgery. Lateral pelvic lymph node dissection was performed only on the side suspected of being positive for LLN metastasis from pretreatment imaging. Adjuvant chemotherapy was considered for all patients, regardless of the pathological results. The regimens used for adjuvant chemotherapy were as follows: Roswell Park regimen of intravenous 5-fluorouracil plus leucovorin, oral tegafur-uracil plus l-leucovorin, oral capecitabine alone, or oral capecitabine plus oxaliplatin.

The pathological tumor response to NACRT was determined based on a grading scale, according to the guidelines of the Japanese Society for Cancer of the Colon and Rectum^17^. This study classified patients with grades 0, 1a, and 1b as poor responders and those with grades 2 and 3 as good responders.

### Definition of mGPS

Peripheral blood (10 mL) samples were collected from all patients 1 week before surgery (postNACRT). Albumin and CRP were obtained from the hospital information system. The mGPS was calculated as follows: patients with elevated levels of CRP (> 1 mg/dL) and hypoalbuminemia (< 3.5 g/dL) were allocated a score of 2, patients with only an abnormal CRP level (> 1 mg/dL) were given a score of 1, and those with a normal CRP level (≤ 1 mg/L) regardless of albumin level were given a score of 0.

### Statistical analysis

Categorical variables were compared using chi-square or the Fisher’s exact test when appropriate. Nonparametric variables were presented as median values, and ranges were compared using the Mann–Whitney U test. Survival analysis was performed using the Kaplan–Meier method, and a univariate survival comparison was performed using the log-rank test. Variables with a p-value < 0.1 in the univariate analysis were further evaluated in the multivariate analysis using the Cox proportional hazard model. All statistical analyses were performed using EZR software (Saitama Medical Center, Jichi Medical University, Saitama, Japan). Statistical significance was set at p < 0.05.

## Results

The patients were divided into high (mGPS = 1 or 2; n = 23) and low (mGPS = 0; n = 50) mGPS groups.

Patient and tumor characteristics are summarized in Table 1. Body mass index was significantly higher in the low mGPS group (23 kg/m^2^ vs. 21 kg/m^2^, p = 0.033). However, the other parameters were comparable between the high and low mGPS groups.

**Table 1:** Patient and tumor characteristics.

	mGPS = 0	mGPS = 1, 2	*P*
	*n* = 50	*n* = 23	
Age, median (range)	67 (39–80)	69 (56–88)	0.418
Sex, n (%)			0.577
Male	15 (30.0)	5 (21.7)	
Female	35 (70.0)	18 (78.3)	
BMI (kg/m^2^), median (range)	23 (15–30)	21 (15–26)	0.033
ASA score, n (%)			0.767
I	21 (42.0)	11 (47.8)	
II	24 (48.0)	9 (39.1)	
III	5 (10.0)	3 (13)	
cT*, n (%)			0.139
is/1	1 (2.0)	1 (4.3)	
2	4 (8.0)	0 (0)	
3	37 (74.0)	14 (60.9)	
4	8 (16.0)	8 (34.8)	
cN*, n (%)			0.190
0	8 (16.0)	5 (21.7)	
1	19 (38.0)	3 (13.0)	
2	23 (46.0)	15 (65.2)	
cStage*, n (%)			0.998
0/I	1 (2.0)	1 (4.3)	
II	7 (14.0)	4 (17.4)	
III	22 (44.0)	10 (43.5)	
IV	20 (40.0)	8 (34.9)	
Completion of NACRT, n (%)			1
Yes	44 (88.0)	20 (87.0)	
No	6 (12.0)	3 (13.0)	
Adjuvant chemotherapy, n (%)			1
Yes	22 (44.0)	10 (43.5)	
No	28 (56.0)	13 (56.5)	

*BMI* body mass index, *ASA* American Society of Anesthesiologists, *NACRT* neoadjuvant chemoradiotherapy.

*Tumors were classified according to the American Joint Committee on Cancer (AJCC) TNM system.

The operative outcomes are shown in Table 2. Abdominoperineal resection was performed more frequently in the high mGPS group, whereas anterior resection was performed more frequently in the low mGPS group. Lateral pelvic lymph node dissection was more frequently performed in the high mGPS group. Operation time was significantly longer in the high mGPS group (527 min vs. 432 min, p = 0.045).

**Table 2:** Operative outcomes.

	mGPS = 0	mGPS = 1, 2	*P*
	*n *= 50	*n* = 23	
Operative procedure, n (%)			0.106
AR	17 (34.0)	2 (8.7)	
ISR	7 (14.0)	1 (4.3)	
APR	26 (52.0)	20 (82.6)	
Hartmann	0 (0)	1 (4.3)	
Surgical approach, n (%)			0.283
Open	14 (28.0)	10 (43.5)	
Laparoscopy	36 (72.0)	13 (56.5)	
D*, n (%)			1
D1	0 (0)	0 (0)	
D2	4 (8.0)	1 (4.3)	
D3	46 (92.0)	22 (95.7)	
LLND, n (%)			0.124
Yes	26 (52.0)	17 (73.9)	
No	24 (48.0)	6 (26.1)	
R0 resection, n (%)			1
Yes	45 (90.0)	21 (91.3)	
No	5 (10.0)	2 (8.7)	
Operation time (min)**	432 (211–1138)	527 (317–1513)	0.045
Estimated blood loss (ml)**	122 (0–5345)	510 (0–5020)	0.137
Blood transfusion, n (%)			0.107
Yes	12 (24.0)	10 (43.5)	
No	38 (76.0)	13 (56.5)	

*AR* anterior resection*, ISR* intersphincteric resection*, APR* abdominoperineal resection*, LLND* lateral pelvic lymph node dissection.

*According to the Japanese Classification of Colorectal, Appendiceal, and Anal carcinoma.

**The data are expressed as the median (range).

The postoperative outcomes are shown in Table 3. The rate of postoperative complications was higher in the high mGPS group, although the difference was not statistically significant. The postoperative hospital stay was significantly longer in the high mGPS group (44 vs. 33 days, p = 0.031).

**Table 3:** Postoperative outcomes.

	mGPS = 0	mGPS = 1, 2	*P*
	*n* = 50	*n* = 23	
Postoperative complications (CD ≥ II), n (%)	22 (44.0)	14 (69.1)	0.214
Wound infection	1 (2.0)	1 (4.3)	1
Wound dehiscence	0 (0)	2 (8.6)	0.102
Anastomotic leakage	3 (6.0)	2 (8.6)	1
Bowel obstruction	4 (8.0)	2 (8.6)	1
Lymphorrhea	4 (8.0)	2 (8.6)	1
Deep vein thrombosis	1 (2.0)	0 (0)	1
Dysuria	4 (8.0)	3 (12.9)	0.671
Ureteric injury	1 (2.0)	0 (0)	1
Others	4 (8.0)	2 (8.6)	1
Postoperative complications (CD ≥ III), n (%)	12 (24.0)	10 (43.5)	0.107
Postoperative hospital stay*, days (range)	33 (12–204)	44 (18–205)	0.031
Re-operation within 30 days, n (%)	0 (0)	0 (0)	1
Mortality within 30 days, n (%)	0 (0)	0 (0)	1

*CD* Clavien-Dindo classification.

*The data are expressed as the median (range).

Pathological outcomes are presented in Table 4. Undifferentiated adenocarcinoma was significantly more frequent in the high mGPS group (21.7% vs. 4.0%, p = 0.029). Other factors were comparable between the groups. The pathological responses to NACRT were similar between the groups.

**Table 4:** Pathological outcomes.

	mGPS = 0	mGPS = 1, 2	*P*
	*n* = 50	*n* = 23	
Histological type, n (%)			0.029
Well/moderately	48 (96.0)	18 (78.3)	
Mucinous/poorly	2 (4.0)	5 (21.7)	
ypT*, n (%)			0.203
0/is	3 (6.0)	3 (13.0)	
1	3 (6.0)	0 (0)	
2	12 (24.0)	4 (17.4)	
3	31 (62.0)	13 (56.5)	
4	1 (2.0)	3 (13.0)	
ypN*, n (%)			0.999
0	32 (64.0)	14 (60.9)	
1	9 (18.0)	5 (21.7)	
2	9 (18.0)	4 (17.4)	
ypStage*, n (%)			1
0	5 (10.0)	2 (8.7)	
I	9 (18.0)	3 (13.0)	
II	18 (36.0)	9 (39.1)	
III	13 (26.0)	7 (30.4)	
IV	5 (10.0)	2 (8.7)	
Lymphatic invasion, n (%)			0.558
Absent	40 (80.0)	17 (73.9)	
Present	10 (20.0)	6 (26.1)	
Vascular invasion, n (%)			0.801
Absent	28 (56.0)	14 (60.9)	
Present	22 (44.0)	9 (39.1)	
Histological response**, n (%)			1
Poor (Grade 1a, 1b)	27 (54.0)	13 (56.5)	
Good (Grade 2, 3)	23 (46.0)	10 (43.5)	

*Tumors were classified according to the American Joint Committee on Cancer (AJCC) TNM system.

**According to the Japanese Society for Cancer of the Colon and Rectum guidelines.

**Figure 1:**
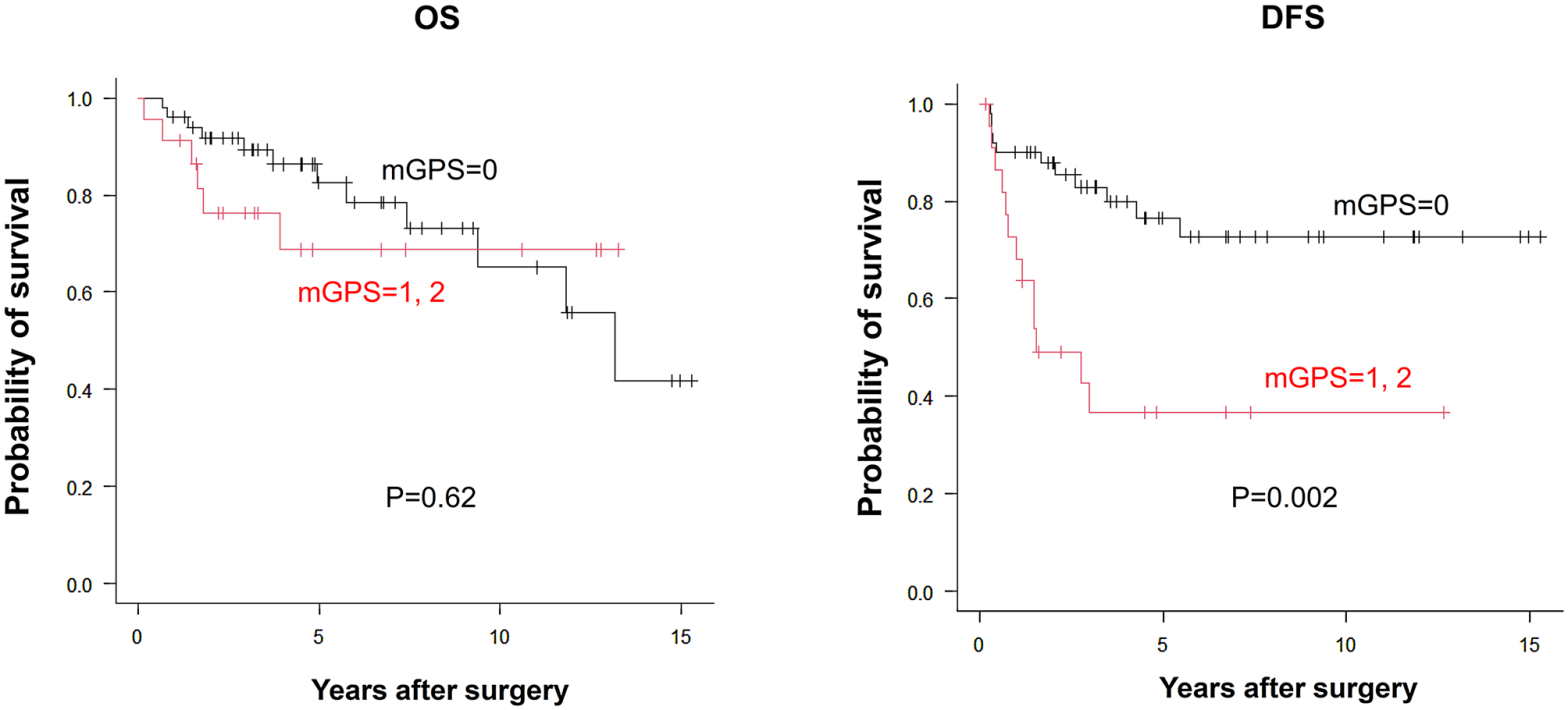
Overall survival (**A**) and disease-free survival curves (**B**) of patients with low mGPS (n = 50) and high mGPS (n = 23). *mGPS* modified Glasgow prognostic score.

The Kaplan–Meier curves for OS and DFS are shown in Fig. 1. The median follow-up period was 49 months. The 5-year DFS was significantly lower in the high mGPS group than that in the low mGPS group (36.7% vs. 76.6%, p = 0.002). In contrast, the 5-year OS was comparable between the groups (68.0% vs. 82.6%, p = 0.62).

Univariate and multivariate analyses of DFS were performed to evaluate risk factors for recurrence (Table 5). In multivariate analysis, high mGPS, poor response to NACRT, and positive pN were significant risk factors. High mGPS was the most significant predictor (p < 0.001).

**Table 5:** Univariate and multivariate analyses for relapse-free survival.

Factor	Total	Univariate	*P*	Multivariate	*P*
		HR (95% CI)		HR (95% CI)	
Age			0.512		
> 70	23	0.73(0.29–1.85)			
≤ 70	50	1			
Sex			0.130		
Female	20	0.44 (0.14–1.27)			
Male	53	1			
mGPS			0.001		< 0.001
1, 2	23	3.68 (1.63–8.26)		5.91 (2.36–14.8)	
0	50	1		1	
Complication ≥ CD grade3			0.319		
Present	22	1.52 (0.66–3.48)			
Absent	51	1			
Pathological response*			0.048		0.023
Poor (Grade 1a, 1b)	40	2.44 (1.00–5.87)		2.93 (1.15–7.4)	
Good (Grade 2, 3)	33	1		1	
pT**			0.335		
3, 4	48	1.54 (0.63–3.72)			
1, 2	25	1			
pN**			0.012		0.005
Positive	27	2.83 (1.25–6.38)		3.38 (1.42–7.99)	
Negative	46	1		1	
Ly			0.379		
Present	16	1.49 (0.61–3.58)			
Absent	57	1			
Histology			0.817		
por/muc	7	0.84 (0.19–3.5)			
Well/mode	66	1			
Adjuvant chemotherapy			0.974		
Yes	32	1.01 (0.45–2.26)			
No	41	1			

*HR* hazard ratio, *CI* confidence interval, *CD* Clavien-Dindo classification.

*According to the Japanese Society for Cancer of the Colon and Rectum guidelines.

**Tumors were classified according to the American Joint Committee on Cancer (AJCC) TNM system.

## Discussion

The GPS was first proposed in 2003 by Forrest et al.^18^ Briefly, patients with an elevated CRP (> 1.0 mg/dL) and hypoalbuminemia (< 3.5 g/dL) scored 2 points. Patients with whom only one of these biochemical abnormalities had a score of 1. Patients in whom neither of these abnormalities were present scored 0. They demonstrated that the GPS is significantly associated with prognosis in patients with inoperable nonsmall-cell lung cancer. McMillan et al. modified the GPS to include patients with hypoalbuminemia but without an elevated CRP score of 0^12^. They found that the mGPS was significantly associated with overall and cancer-specific survival in patients with CRC undergoing resection. Since their report, several studies have demonstrated a significant correlation between the mGPS and prognosis of patients with different cancer types^19–23^. In the present study, we investigated the prognostic significance of the mGPS in patients with LARC who underwent NACRT. To the best of our knowledge, this is the first study to demonstrate that the mGPS is a reliable predictor of oncological outcomes in these patients.

Various markers of inflammation and/or nutritional status might be associated with oncological outcomes in these patients. We evaluated the prognostic nutritional index (PNI), neutrophil-to-lymphocyte ratio, platelet-to-lymphocyte ratio, CRP–albumin ratio, and controlled nutritional status score as possible predictors of oncological outcomes (data not shown). However, only the mGPS was found to be a significant predictor of DFS. We also assessed the mGPS before NACRT and examined its association with potential oncological outcomes. Although the Kaplan–Meier curves for DFS tended to be superior in the low preNACRT mGPS group, the difference did not reach statistical significance (p = 0.07, data not shown). Abe et al. reported that postNACRT, but not preNACRT, malnutrition and sarcopenia were associated with reduced DFS and OS in a similar setting^24^. In this study, we identified postNACRT, but not preNACRT, mGPS as a significant predictor of oncological outcomes in patients with LARC undergoing NACRT.

The mGPS was the most significant predictor of DFS after multivariate analysis in this study, although pathological response to NACRT and pN status were also significant predictors. There was no association between mGPS and pathological response or pN, suggesting that mGPS is an independent predictor of DFS, and that the immuno-nutritional status after NACRT might be more important than expected. Importantly, only the mGPS was available before surgery. Therefore, additional treatments, such as consolidation chemotherapy for patients with a high mGPS before surgery, might be an effective treatment option.

The high mGPS (mGPS = 1 or 2) was significantly associated with lower BMI and more undifferentiated adenocarcinoma in this study. Importantly, several investigators reported that higher BMI might predict better oncological outcomes in rectal cancer patients undergoing NACRT^25–27^. Abdel-Rahman pointed out that a possible link between lower BMI and worse oncological outcomes in patients with advanced colorectal cancer might lie in cancer cachexia^26^. Undifferentiated adenocarcinoma is well known to be associated with poorer outcomes in patients with colorectal cancer than differentiated type. These factors may affect our findings that the higher mGPS was significantly associated with worse prognosis in patients with LARC undergoing NACRT in this study.

Although DFS was significantly worse in the high mGPS group, OS was similar between the groups. One of the possible reasons for this might be that most patients with recurrence in the high mGPS group could receive secondary therapy including surgical resection, chemotherapy, and particle therapy. Peritoneal recurrence developed only in 2 patients. Another reason may be that there were twice as many patients who died of other disease in the low mGPS group as the high mGPS group.

Our results also imply a possible intervention in the immune-nutritional status of patients during NACRT. During preoperative treatment, some patients develop malnutrition owing to high-grade NACRT-induced gastrointestinal toxicities. Furthermore, Lee et al. reported that a decrease in PNI during NACRT was a significant predictor of poor oncological outcomes in patients with LARC^28^. Several antiinflammatory agents have been explored to improve inflammatory and nutritional status^29^. Daily aspirin administration was shown to prevent the incidence of CRC, death, and recurrence^30,31^. A recent meta-analysis showed that NACRT combined with aspirin was more effective than NACRT alone in improving the prognosis of patients with rectal cancer^32^. Statin therapy, both before and after elective surgery for colon cancer, has been reported to reduce all-cause and cancer-specific mortalities^33^. Deva et al. demonstrated that the adjuvant use of histamine 2 receptor antagonists resulted in significantly improved OS in patients with CRC^34^. However, Wong et al. evaluated the effects of preoperative oral supplementation in patients undergoing elective surgery for breast cancer and CRC and demonstrated that it had modest benefits in attenuating weight loss^35^. Importantly, among 73 patients in this study, there were 13 patients with the high mGPS before NACRT but the low mGPS after NACRT, while seven patients with the low mGPS before NACRT but the high mGPS after NACRT. In the remaining patients, the mGPS did not change during NACRT. These findings suggest that the nutritional or inflammatory status can ameliorate or deteriorate during NACRT in a significant number of patients and that its change might affect the oncological outcomes. Therefore, a novel strategy to effectively improve the inflammation and nutritional status during NACRT needs to be established.

This study had some limitations. First, it was a small-scale retrospective study performed at a single institution. Second, potential confounding factors affecting serum albumin and CRP levels, such as infections and autoimmune diseases, were not assessed. Third, the NACRT regimens changed during the study period. In the early period, 45 Gy radiotherapy and oral UFT plus l-LV were employed, whereas in the late period, 50.4 Gy radiotherapy and oral capecitabine were delivered. Further large-scale studies using the same regimens are necessary to draw more definitive conclusions.

## Conclusions

In conclusion, postNACRT mGPS is a significant predictor of DFS in patients with LARC. Therefore, evaluation of the inflammatory and nutritional status during NACRT may be important to improve the oncological outcomes of such patients.

## Data Availability

The datasets used and analyzed during the current study are available from the corresponding author on reasonable request.
